# A Cry for Help Through the Skin: A Case of Dermatitis Artefacta

**DOI:** 10.1192/j.eurpsy.2026.11055

**Published:** 2026-07-31

**Authors:** F. Karsavurdan, O. Metin

**Affiliations:** Psychiatry, Akdeniz University Faculty of Medicine, Antalya, Türkiye

## Abstract

**Introduction:**

Dermatitis artefacta (DA) is a psychodermatological condition involving self-inflicted skin lesions, typically produced without conscious awareness. Unlike malingering or factitious disorder, patients with DA usually deny responsibility for their lesions and often seek dermatological, rather than psychiatric, care. The lesions are typically bizarre in morphology, located on easily accessible areas, and often improve with occlusion. Affective indifference and nonspecific histopathological findings are common. Diagnosis is clinical and requires exclusion of both dermatological and psychiatric differential diagnoses. DA has been associated with borderline personality disorder, post-traumatic stress disorder (PTSD), and depression. The behavior may reflect dissociated affect regulation and serve as a nonverbal cry for help.

**Objectives:**

To present a DA case and emphasize its psychiatric features and diagnostic challenges.

**Methods:**

Clinical case report and brief literature review.

**Results:**

A 30-year-old married woman with two children was referred by dermatology due to multiple sharply demarcated, atrophic facial erosions and prurigo-like papules on both pretibial regions. Lesions had appeared six months earlier and progressively worsened despite extensive dermatological treatment. Upon psychiatric evaluation, the patient was disengaged and emotionally indifferent when questioned about the lesions. She described her internal state as “like a living dead,” reporting pervasive anhedonia, psychomotor retardation, and emotional exhaustion. She disclosed chronic physical and emotional abuse by her spouse and ongoing emotional neglect from her mother. Significant caregiver burden, guilt, and affective dysregulation were also noted.

She later partially admitted to repetitive fingernail manipulation during wakefulness, performed in a dissociative or automatic manner, lacking self-soothing intent. A diagnosis of dermatitis artefacta was considered. Sertraline 50 mg/day was prescribed; however, the patient did not return for follow-up and was lost to psychiatric care despite recommendations for close monitoring.

**Conclusions:**

Dermatitis artefacta is likely underdiagnosed, primarily due to patients’ limited insight and the use of dissociative defense mechanisms. Reported prevalence ranges from approximately 2% to 33%. Although the diagnosis may be suspected and patients referred to psychiatry, individuals with DA, as observed in our case, often avoid or resist psychiatric involvement. To reduce resistance and enhance engagement, a sensitive, nonjudgmental, and multidisciplinary approach—integrating both dermatological and psychiatric care—is essential.

**Disclosure of Interest:**

None Declared

